# The efficacy and safety of submucosal tunnel endoscopic resection for the treatment of upper gastrointestinal submucosal tumors: a systematic review and meta-analysis

**DOI:** 10.3389/fonc.2025.1584205

**Published:** 2025-08-07

**Authors:** Hong Liu, Qing Ma, Linlin Zhu

**Affiliations:** General Practice Ward/International Medical Center Ward, General Practice Medical Center, West China Hospital, Sichuan University, Chengdu, Sichuan, China

**Keywords:** meta-analysis, submucosal tunnel endoscopic resection, upper gastrointestinal submucosal tumors, STER, SMTs

## Abstract

**Objective:**

The comprehensive systematic review was conducted to assess the efficacy and associated factors of submucosal tunnel endoscopic resection (STER) in the treatment of upper gastrointestinal submucosal tumor.

**Methods:**

Databases including PubMed, Web of Science, CNKI, Wang Fang, VIP and Embase were electronically searched for studies on STER for the treatment of upper gastrointestinal submucosal tumors from inception to September 17, 2024. Two authors conducted the literature search independently. A third author resolved any inconsistencies raised between the two. Keywords were used for retrieval, and Boolean operators were used accordingly. The literature on treatment with STER and disease with upper gastrointestinal submucosal tumors will be included in the study. Statistical analysis was performed using Stata 16 software. χ2 test was used to analyze the heterogeneity among the studies. The fixed effects model and random effects model were used for meta-analysis. Standardized Mean Difference (SMD), Relative Risk (RR), and 95% confidence intervals (CI) were used to estimate clinical efficacy/effectiveness. Funnel plot symmetry was used to assess the risk of publication bias between studies.

**Results:**

Eight retrospective studies were ultimately incorporated into the meta-analysis. The experimental group was treated with STER. The control groups included patients receiving conventional endoscopic treatments such as ESD, EFTR, ESE, or LECS. The results of this analysis indicated no statistically significant differences between the intervention group and the control group in complete removal rates [RR=0.98 (0.94, 1.03), P>0.01], mean hospital stay [SMD=-0.40 (-0.89, 0.09), P>0.01], mean operation time [SMD=0.08 (-0.40, 0.57), P>0.01], or complication rates [RR=0.91 (0.44, 1.90), P>0.01]. Subgroup analysis identified age and tumor sizes as sources of heterogeneity in complication rates. In patients older than 55 years, STER exhibited a significantly lower risk of complications compared to traditional treatment modalities, with a risk ratio of 0.151 (95% CI: 0.041–0.558; P < 0.05). This indicates that STER may be a particularly beneficial option for this patient demographic.

**Conclusions:**

The study found no significant differences in complete removal rate, mean hospital stay, mean operation time, and complication rate between STER and Traditional treatment. Nonetheless, a subgroup analysis of patients aged 55 and older uncovered a notable reduction in the incidence of complications among individuals undergoing STER for upper gastrointestinal submucosal tumors in comparison to the control group. These findings suggest that STER may constitute a more advantageous treatment option for elderly patients owing to its lower incidence of complications. There was no evidence of publication bias in the included literature, and the results demonstrated robustness following sensitivity analysis.

## Introduction

Submucosal tumors (SMTs) of the upper gastrointestinal (GI) tract represent a heterogeneous group of lesions originating from the deeper layers of the GI wall, including the muscularis mucosae, submucosa, or muscularis propria. These lesions encompass a variety of pathological entities, such as gastrointestinal stromal tumors (GISTs), leiomyomas, lipomas, and neuroendocrine tumors, each exhibiting distinct biological behavior and potential for malignancy (1). SMTs are often asymptomatic and discovered incidentally during endoscopic or imaging examinations; however, symptomatic cases may present with bleeding, obstruction, or perforation, significantly impairing patient quality of life.

The precise prevalence of upper GI SMTs remains uncertain due to their asymptomatic nature and underdiagnosis, but studies estimate an incidence of approximately 0.4%–1% in routine upper endoscopic evaluations (2). Despite their relative rarity, the potential for malignant transformation, particularly in GISTs, underscores the importance of early detection and appropriate management. Malignant SMTs, if left untreated, can lead to serious complications such as metastasis, upper GI bleeding, and significant morbidity, which pose a substantial threat to patient survival (3).

Beyond individual health concerns, SMTs impose a considerable socioeconomic burden. The healthcare costs associated with diagnostic procedures, follow-up imaging, and therapeutic interventions are substantial. Furthermore, the progression of malignant lesions may lead to loss of productivity and long-term care needs, creating a ripple effect on societal economic resources (4). Therefore, early treatment and intervention of upper gastrointestinal submucosal tumors are urgent.

At present, surgical resection and endoscopic resection represent two viable treatment options for upper Gastrointestinal Submucosal Tumor. Compared to surgical resection, endoscopic resection offers the advantages of minimal invasiveness and a shortened postoperative recovery period, leading to its increasing adoption in clinical practice and widespread recognition. Endoscopic submucosal dissection (ESD), endoscopic submucosal excision (ESE), and endoscopic full-thickness resection (EFR) have demonstrated safety and efficacy in the treatment of these tumors. However, these techniques often compromise mucosal integrity, elevating the risk of perforation, infection, and postoperative stenosis (1, 2, 5, 6) For instance, ESD of SMTs within the superficial submucosa is generally feasible; however, SMTs located in deeper layers frequently result in perforation during the procedure (3, 7). Additionally, esophageal ESD is associated with a risk of postoperative strictures, particularly when large circumferential areas are resected. Stricture rates can be as high as 17% to 88% in cases involving more than three-quarters of the esophageal circumference (8). EFR necessitates extensive excision, resulting in larger defects and a higher risk of complications, with studies reporting an adverse event rate of 18.6%, including bleeding and perforation (9). Furthermore, Endoscopic Mucosal Resection (EMR) is limited to lesions ≤20 mm that can be adequately lifted, with reduced efficacy in larger or fibrotic lesions (10). These limitations highlight the need for tailored endoscopic techniques to optimize outcomes.

Submucosal tunneling endoscopic resection (STER) has been increasingly adopted in clinical practice, particularly in China, for the management of gastrointestinal SMTs originating from the muscularis propria layer. However, despite its growing popularity, comprehensive evidence evaluating its efficacy and safety remains limited (1). The technique minimizes bleeding by allowing precise visualization of the vascular network and reduces tissue damage by avoiding manipulation of mediastinal structures during esophageal procedures (11). Although technically challenging, STER is safe for use in the stomach, rectum, and esophagus, offering an effective and minimally invasive approach to SMT management (12–14).

STER has shown significant promise in managing upper Gastrointestinal SMT, particularly those originating from the muscularis propria. Its advantages include high en bloc (94.6%) and complete resection rates (97.5%) and the preservation of mucosal integrity, reducing risks of perforation and postoperative complications (15).However, existing studies on SMT treatment are limited by small sample sizes, short follow-up durations, and methodological heterogeneity, which hinder generalizability and long-term outcome assessment.

Consequently, we sought to comprehensively evaluate the overall efficacy of STER in the treatment of upper Gastrointestinal SMT through a meta-analysis and systematic synthesis analysis. Our objective was to furnish evidence-based recommendations and guidelines to inform clinical practice in the treatment of upper Gastrointestinal SMT.

## Methodology

### Search strategies

This study was based on a systematic review and meta-analysis of the Preferred Reporting Project (PRISMA) report list. Articles were systematically searched from electronic databases such as PubMed, Web of science, CNKI, WanFang, vip, Embase, etc. Search for studies published in English as of September 17, 2024. Two authors (Hong Liu and Qing Ma) conducted the article search independently. The third author (Linlin Zhu) addresses the inconsistencies that arise between the two authors. Search terms are listed as follows: submucosal tumor, submucosal lesions, STMs, Upper gastrointestinal SMT, STER.

### Inclusion and exclusion criteria

Inclusion criteria:

1. Inclusion subjects were upper gastrointestinal SMT;
2. The treatment method was STER;
3. The article has clear outcome indicators and other data, such as Complete removal rate, mean hospital stay, mean operation time, and complication rate;
4. The article has basic demographic information: basic information of the included population, publication year, treatment method;
5. Study design must be retrospective with a control group;
6. The tumor must originate from or involve the muscularis propria layer, as confirmed by endoscopic ultrasonography or imaging.

Exclusion Criteria:

1. The subjects were not upper gastrointestinal SMT;
2. The treatment method is not STER;
3. The types of articles are conference, meta, review, case report;
4. The main outcome indicators were missing;
5. Article Treatment methods without control group.

### Data extraction

All retrieved studies are imported into the EndNote X9 software. Duplicate publications and data reuse were excluded from this study, and the three authors independently screened titles and abstracts to identify eligible studies. Extract the full text and read the articles that meet the inclusion criteria to determine if they make it into the final analysis. The author identifies any conflicts through discussion or negotiation with the fourth member of the review team.

Two analyzers (Hong Liu and Linlin Zhu) independently screened the demographic and intervention information from the original literature. The extracted information and data are as follows: (1) name of the first author; (2) type of study; (3) region of author; (4) sample size; (6) Type of disease, and treatment mode; (5) age and female/male ratio of experimental participants; (6) Year of study released; (8) Intervention measures (7) outcome indicators complete removal rate, mean hospital stay, mean operation time, and complication rate. Outcome indicators were defined as follows: ollows:r removal: refers to successful en bloc resection without residual lesion; esion;lnlon; rateo includes intraoperative or postoperative adverse events such as bleeding, perforation, and infection.

### Quality assessment

Critical Appraisal Skills Program(CASP), as a standard for literature quality evaluation, mainly includes 11 questions. The first three questions are screening questions and can be answered quickly. If the answer to both is yes”, it is worth proceeding with the remaining questions. There is some degree of overlap between the questions, we are asked to record a yes”, noor can’t tellto most of the questions. A number of italicized prompts are given after each question. Each question on the CASP scale is answered with yes”, no”, or can’t tell”. Each yesgets 1 point, and Noand can’t tellget 0 points. Scoring criteria: Score more than 70% is high quality; A score of 50%-70% is considered medium quality; A score below 50% is considered low quality.

### Risk of publication bias

The risk of publication bias between studies was assessed using funnel plot symmetry.

### Data analysis

Statistical analysis was performed using Stata 16 software. χ2 test was used to analyze the heterogeneity among the studies. If *P* > 0.1 and *I^2^* < 50%, there was no statistical heterogeneity among the studies. The fixed effect model was used for meta-analysis. On the contrary, it indicates that there is heterogeneity among different studies. The random effects model is selected, and the source of heterogeneity is further discussed by subgroup analysis. If no source of heterogeneity could be found, descriptive analysis was used. Sensitivity analysis was used to evaluate the robustness of the results. SMD, RR, and 95% CI were used to estimate clinical efficacy. Funnel plot asymmetry was assessed using inverted funnel diagrams to evaluate potential publication bias.

## Results

### Selected studies

**Figure 1:**
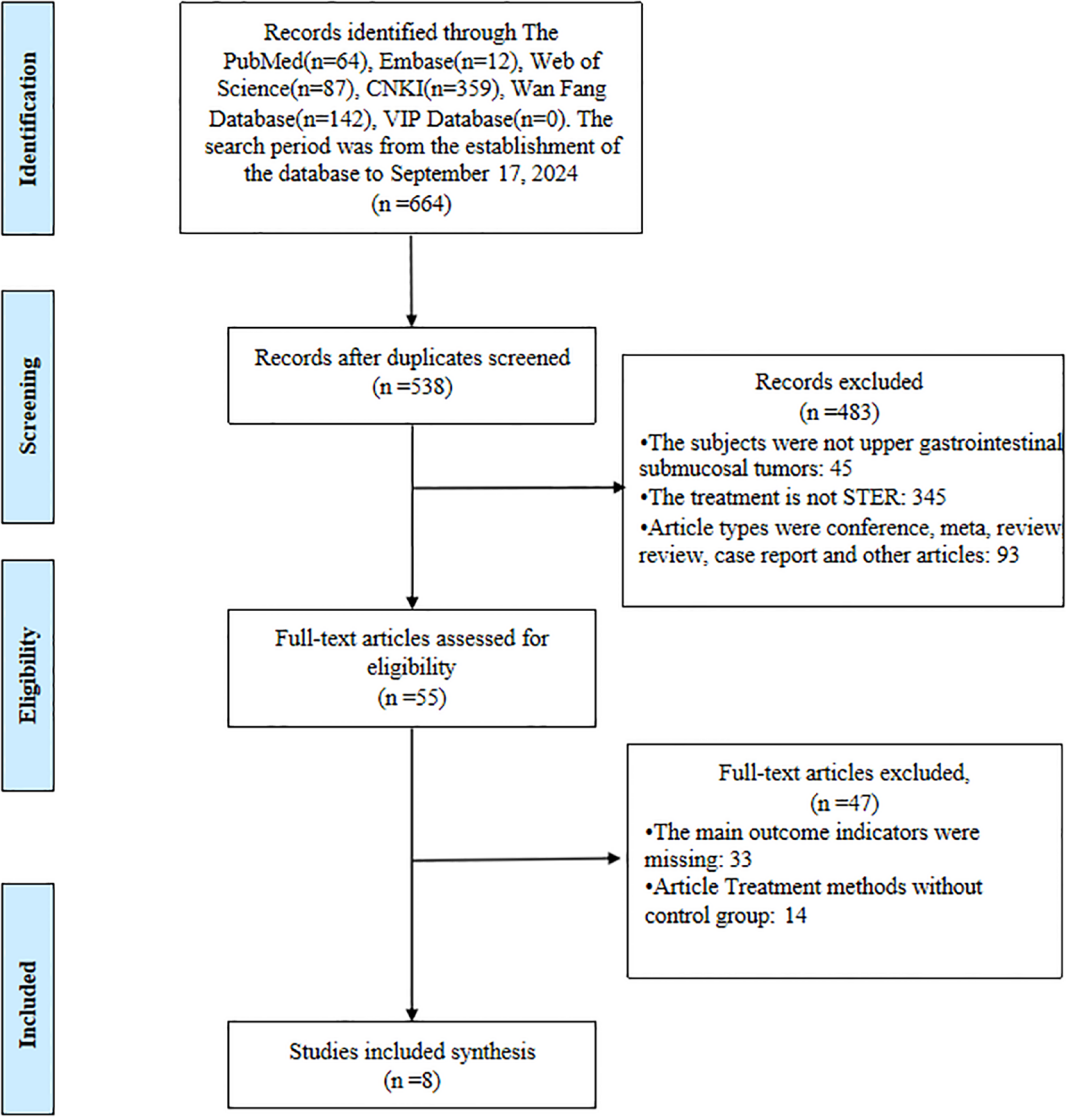
The literature screening process of the meta-analysis.

A systematic and stepwise approach was employed to identify eligible studies. A total of 664 articles were retrieved from all databases. First, all the studies identified from the entire search were exported to EndNote X9 Citation Manager, and 126 duplicate articles were deleted. We excluded 483 articles by title and abstract. Following a thorough full-text evaluation, an additional 47 articles were deemed ineligible. Ultimately, 8 articles met all inclusion criteria and were included in the final analysis. Details of the selection process are shown in **Figure 1**.

### Study characteristics

The basic characteristics of the included literature are shown in **Table 1**. All the 8 literature are retrospective studies, and all of them are from China. The inclusion and exclusion criteria and the evaluation criteria for each indicator are detailed in **Supplementary Table 1**.

**Table 1:** Baseline characteristics of included studies.

First Author	Year	Country	Disease type	Total number			Tumor size(mm)		treatment mode		Gender (males)		Age(year)				Mean operating time (min)				The median length of stay after surgery(day)				Complete removal		Number of complications	
				N	Exp	Con	Exp	Con	Exp	Con	Exp	Con	Exp		Con		Exp		Con		Exp		Con		Exp	Con	Exp	Con
													Mean	sd	Mean	sd	Mean	sd	Mean	sd	Mean	sd	Mean	sd				
Michel Kahaleh (16)	2022	China	GISTs	68	34	34	23.2 ± 18.2	35.7 ± 15.8	STER	EFTR	19	14	59.3	13.3	65	16.3	96.2	52	87.6	40.7	1.5	0.78	1.8	1	NA	NA	1	3
Michel Kahaleh (16)	2022	China	GISTs	62	34	28	23.2 ± 18.2	33.4 ± 16.7	STER	LECS	19	12	59.3	13.3	61.5	11	96.2	52	128.7	48.6	1.5	0.78	2.3	0.71	NA	NA	1	7
Philip Wai Yan Chiu (2)	2022	China	GISTs	46	20	26	20.9 ± 12.4	22.0 ± 9.79	STER	EFTR	8	8	57.5	NA	59.6	NA	94.9	NA	84.9	NA	4.38	NA	3.5	NA	16	26	NA	NA
Luo Yingshu (17)	2021	China	SMTs	95	26	69	11.2	NA	STER	ESD	NA	NA	54.19	9.825	NA	NA	84.45	NA	51.3	NA	14.42	NA	8.9	NA	21	59	5	4
Luo Yingshu	2021	China	SMTs	143	26	117	11.2	NA	STER	ESE	NA	NA	54.19	9.825	NA	NA	84.45	NA	55.19	NA	14.42	NA	9.48	NA	21	111	5	6
Luo Yingshu (17)	2021	China	SMTs	54	26	28	11.2	NA	STER	EFTR	NA	NA	54.19	9.825	NA	NA	84.45	NA	68.36	NA	14.42	NA	11.36	NA	21	25	5	2
Zou Huan (4)	2022	China	GISTs	31	6	25	31 ± 0.9	41.2 ± 5.1	STER	ESD	28	14	58.25	10.62	58.24	2.28	NA	NA	NA	NA	NA	NA	NA	NA	6	23	0	4
Zou Huan (4)	2022	China	GISTs	26	6	20	31.0 ± 0.9	40.5 ± 3.9	STER	EFTR	28	10	58.25	10.62	58.9	2.43	NA	NA	NA	NA	NA	NA	NA	NA	6	20	0	20
Tu Sufang (18)	NA	China	SMTs	167	148	19	20.5 ± 10.4	32.2 ± 12.2	STER	ITW-STER	NA	NA	48.6	NA	NA	NA	46.8	24.9	58.9	23	NA	NA	NA	NA	148	19	9	0
Lin Liangdou (19)	2018	China	UGIST	86	41	45	12.36 ± 7.38	12.07 ± 8.67	STER	ESD	12	13	49.8	11.62	53.03	13.83	60	20	60	20	6.39	1.55	7.1	1.72	39	43	17	21
Liu Ying (20)	2014	China	SMTs	31	14	17	1.3 ± 0.5	1.2 ± 0.5	STER	ESD	6	10	51	9	52	12	95	27	61	14	7.6	1	7.1	1.6	13	13	2	5
Yuyong Tan (21)	2016	China	GISTs	52	20	32	17.8 ± 7.2	15.4 ± 6.6	STER	EFTR	8	13	51.3	7.9	54.1	9.6	74.9	32.1	69.1	27	NA	NA	NA	NA	19	31	1	5

STER, submucosal tunnel endoscopic resection; ESE, Endoscopic Submucosal Excision; EFTR, Endoscopic Full-Thickness Resection; LECS, Laparoscopic Endoscopic Cooperative Surgery; ESD, Endoscopic Submucosal Dissection; EMR, Endoscopic Mucosal Resection; ITW-STER, Improved tunnel window method STER; GISTs, Gastrointestinal Stromal Tumors; SMTs, Submucosal tumors; UGIST, Upper gastrointestinal submucosal tumors; N, Total Number; Exp, Experimental group; Con, Control group

### Quality assessment

The quality assessment of this study is shown in **Supplementary Table 2**. Through literature quality assessment, a total of 6 high-quality studies, 1 medium-quality study and 1 low-quality study were included.

### Results of index meta-analysis

#### Complete removal rate

**Figure 2:**
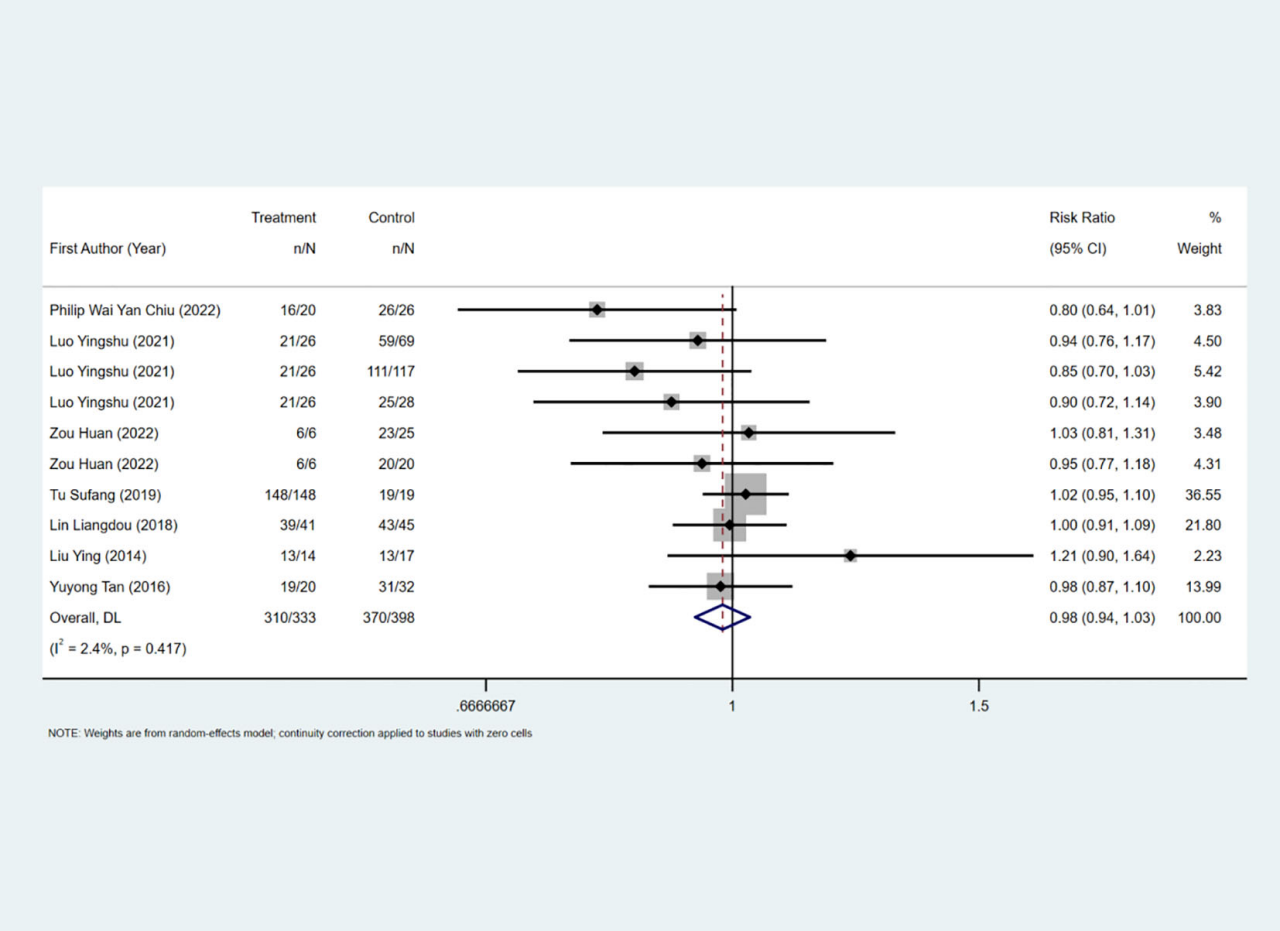
Complete removal rate forest map.

The complete removal rate was reported in seven studies, and the results of the inter-study heterogeneity test were *P>0.1, I^2^ = 2.4%*. The fixed effect model was used for meta-analysis, and there was no statistical difference in the complete removal rate [*RR=0.98 (0.94, 1.03), P>0.01*] between the two groups. (**Figure 2**, **Table 2**). The treatment methods of the experimental group were STER, and the treatment methods of the control group included ESD, improved STER and EFTR.

**Table 2:** Effect size.

Index	Heterogeneity test		Pooled RR(95%CI)/ SMD(95%CI)	P
	P	I^2^		
Mean operating time	<0.001	80.8%	0.085 (-0.404,0.574)	0.734
Median length of stay	0.016	71.1%	-0.403 (-0.892,0.086)	0.106
Complete removal rate	0.417	2.4%	0.984 (0.940,1.029)	0.479
Complications rate	0.008	59.6%	0.912 (0.438,1.900)	0.806

### Secondary outcome measure

**Figure 3:**
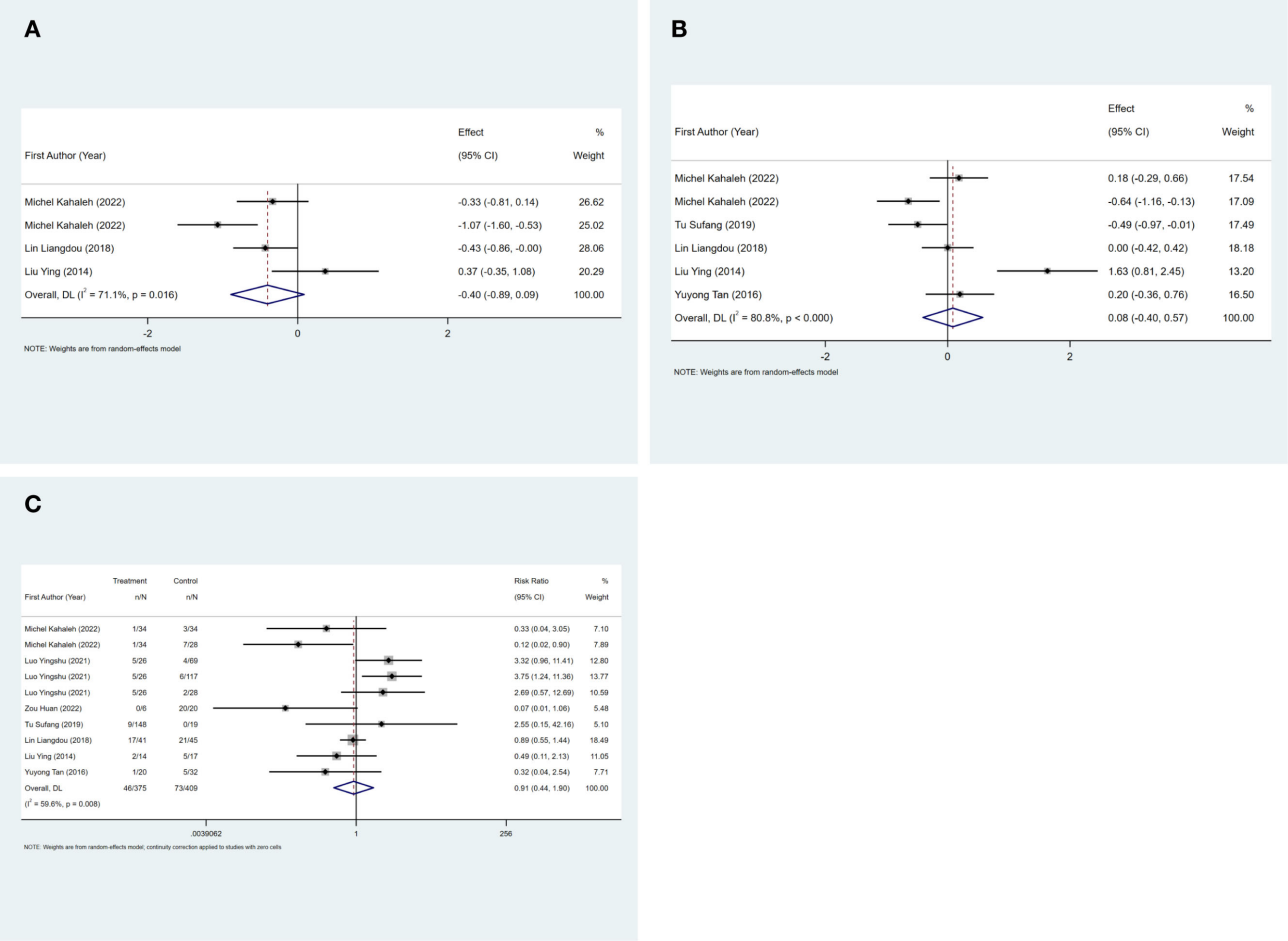
**(A)** Mean hospital stay forest map. FIGURE 3 **(B)** Mean operation time forest map. FIGURE 3 **(C)** Complication rate forest map.

The mean hospital stay was reported in three studies, and the results of the inter-study heterogeneity test were *P=0.016, I^2^ = 71.1%*. The random effect model was used for meta-analysis, and there was no statistical difference [*SMD=-0.40 (-0.89, 0.09), P>0.01*] in mean hospital stay between the two groups. The mean operation time was reported in five studies, and the results of the inter-study heterogeneity test were *P<0.001, I^2^ = 80.8%*. The random effect model was used for meta-analysis, and there was no statistical difference in mean operation time [*SMD=0.08 (-0.40, 0.57), P>0.01*] between the two groups. (**Figures 3A, B**, **Table 2**).The treatment mode of the experimental group was STER, and the treatment mode of the control group included ESD and EFTR

The complication rate was reported in seven studies, and the results of the inter-study heterogeneity test were *P=0.008, I^2^ = 59.6%*. The random effect model was used for meta-analysis, and there was no statistical difference [*RR=0.91 (0.44,1.90), P>0.01*] in complication rate between the two groups. (**Figure 3C**, **Table 2**). The experimental group was STER, and the control group was ESD, EFTR and improved STER.

### Subgroup analysis

Subgroup analyses identified age and tumor sizes as sources of heterogeneity in the complication rate. Treatment modality emerged as a factor influencing mean operation time. Among patients older than 55 years, STER demonstrated significantly lower complication rates compared to alternative therapeutic approaches, suggesting that STER may confer a reduced risk of complications for this patient demographic. (**Table 3**)

**Table 3:** Summary of subgroup analysis results.

Index	Subgroup		Included studies	Heterogeneity test		RR (95%CI)	P
				P	I^2^		
Mean operation time	AGE	>=55	2	0.021	81.4%	-0.224 (-1.035, 0.587)	0.588
		<55	4	0.000	84.5%	0.267 (-0.426, 0.959)	0.45
	Tumor size (mm)	>=20mm	3	0.040	68.20%	-0.311 (-0.812,0.191)	0.224
		<20mm	3	0.002	83.50%	0.541 (-0.286,1.368)	0.200
	Treatment mode	1	1	/	/	-0.644 (-1.157,-0.130)	0.014
		2	2	0.967	0.00%	0.191 (-0.172,0.554)	0.303
		3	2	0.000	91.60%	0.775 (-0.820,2.371)	0.341
		5	1	/	/	-0.490 (-0.970,-0.009)	0.046
Complete removal rate	AGE	>=55	3	0.320	13.50%	0.920 (0.798,1.060)	0.247
		<55	7	0.450	0.00%	0.994 (0.949,1.041)	0.787
	Tumor size (mm)	>=20mm	4	0.240	29.20%	0.975 (0.883,1.076)	0.612
		<20mm	6	0.460	0.00%	0.984 (0.940,1.029)	0.377
	Treatment mode	2	4	0.477	0.00%	0.936 (0.858,1.022)	0.139
		3	4	0.590	0.00%	1.005 (0.930,1.086)	0.901
		4	1	/	/	0.851 (0.702,1.032)	0.101
		5	1	/	/	1.022 (0.952,1.097)	0.543
Complications rate	AGE	>=55	3	0.660	0%	0.151 (0.041,0.558)	0.005
		<55	7	0.052	52%	1.451 (0.720,2.926)	0.298
	Tumor size (mm)	>=20mm	4	0.260	25.70%	0.260 (0.065,1.040)	0.057
		<20mm	6	0.030	59.10%	1.407 (0.663,2.985)	0.374
	Treatment mode	1	1	/	/	0.118 (0.015,0.900)	0.039
		2	4	0.086	54.50%	0.476 (0.102,2.223)	0.345
		3	3	0.090	58.10%	1.118 (0.448,2.791)	0.811
		4	1	/	/	3.750 (1.238,11.358)	0.021
		5	1	/	/	2.550 (0.154,42.160)	0.513

Treatment mode: 1. STER VS LECS; 2. STER VS EFTR; 3. STER VS ESD; 4. STER VS ESE; 5.other.

### Publication bias

**Figure 4:**
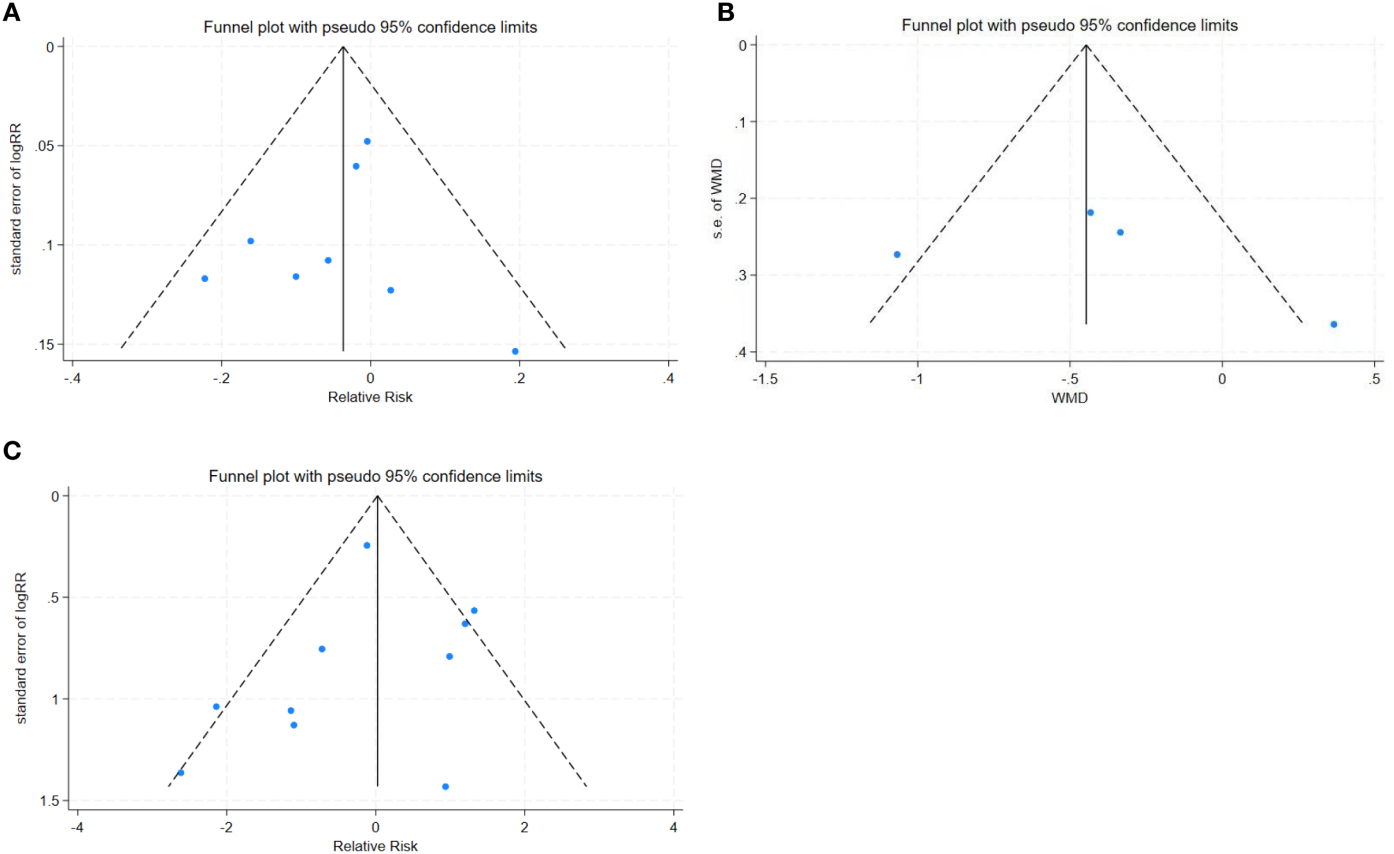
**(A)** Complete removal rate funnel plot. FIGURE 4 **(B)** Mean operation time funnel plot. FIGURE 4 **(C)** Complication rate funnel plot.

Deeks’ funnel plot asymmetry test showed no significant publication bias, the funnel diagram was shown in **Figures 4A–C**.

### Sensitivity analysis

Sensitivity analysis was performed using complete removal rate, mean operation time, and complication rate. The results showed that after deleting all studies one by one, the obtained 95%CI did not cross the nullity line, and the comprehensive effect size point was still within the original 95%CI, indicating that the results of this study had strong robustness. See **Tables 4**–**6**.

**Table 4:** The sensitivity analysis results (mean operation time).

Study omitted	Estimate	95% CI	
Michel Kahaleh (2022) (16)	0.076	-0.524	0.677
Michel Kahaleh (2022) (16)	0.231	-0.291	0.753
Tu Sufang (2019) (18)	0.210	-0.348	0.769
Lin Liangdou (2018) (19)	0.120	-0.508	0.748
Liu Ying (2014) (20)	-0.14	-0.483	0.183
Yuyong Tan (2016) (21)	0.072	-0.511	0.655
Combined	0.084	-0.404	0.574

**Table 5:** The sensitivity analysis results (complete removal rate).

Study omitted	Estimate	95% CI	
Philip Wai Yan Chiu(2022) (2)	0.671	0.356	1.262
Luo Yingshu (2021) (17)	0.514	0.254	1.04
Luo Yingshu (2021) (17)	0.692	0.349	1.371
Luo Yingshu (2021) (17)	0.571	0.296	1.099
Zou Huan (2022) (4)	0.530	0.288	0.979
Zou Huan (2022) (4)	0.559	0.306	1.022
Tu Sufang (2019) (18)	0.559	0.306	1.022
Lin Liangdou (2018) (19)	0.531	0.282	1.000
Liu Ying (2014) (20)	0.449	0.236	0.854
Yuyong Tan (2016) (21)	0.556	0.300	1.032
Combined	0.559	0.300	1.032

**Table 6:** The sensitivity analysis results (complication rate).

Study omitted	Estimate	95% CI	
Michel Kahaleh (2022) (16)	0.834	0.530	1.312
Michel Kahaleh (2022) (16)	0.944	0.593	1.504
Luo Yingshu (2021) (17)	0.671	0.418	1.077
Luo Yingshu (2021) (17)	0.649	0.403	1.044
Luo Yingshu (2021) (17)	0.715	0.449	1.139
Zou Huan (2022) (4)	1.012	0.633	1.618
Tu Sufang (2019) (18)	0.753	0.479	1.182
Lin Liangdou (2018) (19)	0.796	0.474	1.337
Liu Ying (2014) (20)	0.838	0.530	1.325
Yuyong Tan (2016) (21)	0.846	0.536	1.335
combined	0.799	0.513	1.245

## Discussion

This meta-analysis compared STER with conventional surgical approaches for SMTs. While no statistically significant differences were observed in complete resection rate, hospital stay, operative time, or overall complication rate between groups, a subgroup analysis revealed a significantly lower complication rate for STER in patients aged ≥55 years. This finding suggests that STER may offer a safer treatment option for older patients, who often present with comorbidities that increase the risk of postoperative complications. The minimally invasive nature of STER likely contributes to this benefit by facilitating a smoother postoperative recovery.

This study explored the sources of heterogeneity through subgroup analysis. We conducted subgroup analyses on the three outcome indicators of Mean operation time, Complete removal rate, and Complications rate respectively. It was found in the Complications rate analysis that age and tumor size were sources of heterogeneity, but there was no similar conclusion in the Mean operation time analysis. The reasons leading to this conclusion are diverse, including differences in surgical techniques and the experience of surgeons, different patient groups with different tumor characteristics and comorbidities, inconsistent outcome definitions and reporting practices, and differences in postoperative management. This is also one of the limitations of this study. Future research should focus on standardizing surgical protocols, defining outcomes consistently, exploring the impact of patient-specific factors through meta-regression, and using robust methodologies to mitigate these sources of variability and improve the generalizability of findings.

This meta-analysis’s findings largely corroborate existing literature supporting the efficacy and safety of STER for SMTs, particularly regarding reduced postoperative complications. Several studies, such as Tao Chen et al (22), reported low complication rates and minimal recurrence, aligning with our results. Although these studies were not included in our meta-analysis due to lack of control groups or methodological differences, their findings provide contextual support for the observed safety profile of STER. Similarly, Philip Wai Yan Chiu (2) found shorter hospital stays with STER compared to EFTR, a trend also suggested by our analysis, albeit not statistically significant overall. Peter Dellatore (23) similarly found no significant difference in major complications between STER and EFTR, consistent with our findings, although STER showed advantages in procedural time and hospital stay.

However, discrepancies exist. Deepanshu Jain’s study (24)reported higher complication rates with STER. This contrast may be attributed to differences in patient selection criteria (e.g., inclusion of larger tumors) or methodological variations (e.g., different definitions of complications). The observation that many patients in Jain’s study responded well to conservative treatment suggests a potential selection bias towards less severe cases. Furthermore, Yong Lv’s findings (25) highlighting increased complications and operative times with tumors ≥25mm are consistent with our identification of tumor size as a source of heterogeneity in complication rates. This highlights the importance of considering tumor size in patient selection for STER, as larger lesions present challenges for visualization and increased surgical complexity.

The findings of this study underscore the importance of tailoring treatment decisions to individual patient characteristics, particularly age and overall health profile. In patients aged 55 and older, STER appears to be a potentially safer therapeutic option. However, given the limited number of included studies and the wide confidence intervals observed, this finding should be interpreted with caution and considered hypothesis-generating rather than conclusive. To optimize outcomes and minimize complications, healthcare providers should meticulously develop personalized treatment plans. However, this study is subject to certain limitations. The relatively small number of included studies and limited sample size may compromise the robustness of the results. Moreover, the brevity of follow-up periods in some studies precludes a comprehensive evaluation of long-term outcomes. Additionally, variations in surgical techniques and physician expertise may influence the consistency of findings. Future research endeavors should prioritize expanding sample sizes, conducting multicenter studies, and establishing ongoing long-term follow-up protocols. Furthermore, randomized controlled trials are essential for elucidating the efficacy and safety of different surgical modalities across diverse patient populations. Moreover, in-depth investigations into other potential factors influencing postoperative complications are necessary to refine treatment strategies.

Collectively, this meta-analysis underscores the efficacy of STER in the treatment of submucosal tumors, particularly among patients aged 55 and older, demonstrating a favorable safety profile in comparison to traditional surgical interventions. As research into STER’s efficacy continues to evolve, it is anticipated that STER will emerge as a valuable therapeutic option for this condition.

Additionally, lesion size is a critical consideration for STER candidacy. Several studies have suggested that lesions smaller than 30–35 mm are more suitable for STER due to technical feasibility and lower complication risks. For example, Lv et al. reported increased complication rates and prolonged operative time in tumors ≥25 mm (25). Consequently, careful preoperative assessment of lesion size should guide patient selection for optimal safety and outcomes.

### Study limitation

Our investigation of STER for the treatment of SMTs encountered certain limitations. Initially, our findings did not reveal a distinct advantage of STER over alternative treatment modalities in terms of clinical efficacy. This may be attributed to the relatively small sample size, which could potentially amplify the risk of methodological errors and biases in our results.

Secondly, the traditional group in this study included various techniques such as EFTR, ESD, ESE, LECS, etc. This may to some extent affect the accuracy of the results. Since there are relatively few research reports on a single specific technique at present, more traditional technical methods were included in the literature screening. We acknowledge the limitations of this study. In future research, we will expand the scope of the search databases and conduct a more comprehensive search of grey literature.

Upon examination of secondary endpoints, including mean hospital stay, mean operative time, and the incidence of complications, substantial heterogeneity was evident across the included literature. While age, treatment modality, and tumor sizes were identified as potential sources of heterogeneity, the paucity of studies addressing these factors precluded a comprehensive exploration of their influence.

All included studies were retrospective in design, and none were randomized controlled trials. Moreover, the control groups varied significantly in terms of intervention type, introducing potential bias and limiting the generalizability of the findings. These methodological limitations necessitate cautious interpretation of the pooled results.

Given the identified limitations, we propose that future research endeavors should expand the sample size to bolster the reliability and generalizability of the findings. Moreover, a paramount focus should be placed on incorporating high-quality literature to mitigate bias and augment the accuracy of the analysis. By implementing these measures, subsequent studies will be better equipped to comprehensively evaluate the efficacy of STER in the management of submucosal tumors and furnish more robust evidence to inform clinical decision-making.

## Conclusion

The study revealed no significant disparities in complete removal rate, mean hospital stay, mean operation time, and complication rate between STER and alternative treatment modalities. Nevertheless, a subgroup analysis of patients aged 55 and older demonstrated a notable reduction in the incidence of complications among those treated with STER for submucosal tumors compared to the control group. These findings collectively suggest that STER may represent a more advantageous treatment option for elderly patients owing to its lower complication profile. Furthermore, the absence of publication bias within the included literature and the maintenance of stability following sensitivity analysis underscore the robustness of these findings.

## Data Availability

The original contributions presented in the study are included in the article/**Supplementary Material**. Further inquiries can be directed to the corresponding author.
