# Supplementary material for: The efficacy and safety of submucosal tunnel endoscopic resection for the treatment of upper gastrointestinal submucosal tumors: a systematic review and meta-analysis
Source: Front Oncol. 2025 Aug 7;15:1584205. doi: 10.3389/fonc.2025.1584205 (PMC12367509; doi:10.3389/fonc.2025.1584205)
Supplement: Supplementary file 1 [file Table1.docx]

| Supplementary Table 1 | | | | | | | | |
| --- | --- | --- | --- | --- | --- | --- | --- | --- |
| First author | Year of publication | Research design method | Disease type | Case diagnosis | specific treatment steps | | Inclusion criteria | Exclusion criteria |
|  |  |  |  |  | Exp. | Con. |  |  |
| Michel Kahaleh | 2022 | Retrospective study | leiomyoma, gastrointestinal stromal tumors(GISTs) and other | leiomyoma, GIST、ectopic pancreas、lipoma、 schwannoma、carcinoid tumors、and granular cell tumors | NA | NA | NA | NA |
| Philip Wai Yan Chiu | 2022 | Retrospective study | gastrointestinal stromal tumors (GISTs) | GIST | after endoscopic location of the gastric GIST and pre-injection, a 2- to 3-cm mucosal entrance was created 2 cm proximal to the tumor border. A short submucosal tunnel was developed up to the point of visualization of the tumor. The lat eral borders of the subepithelial tumor were dissected, and subsequently a 2-cm pocket was created distally for manipula tion. Subsequently, the tumor was dissected away from muscu laris propria layer to the subserosal layer. Upon completion of the dissection, the tumor was retrieved per orally. The mucosal entrance was closed by primary clip closure method. |  | Patients with gastric GISTs < than 4 cm were included. Clinical outcomes | NA |
| Luo Yingshu | 2021 | Retrospective study | Submucosal tumors (SMTs) | Leiomyoma, stromal tumor | NA | NA | The patient was found to have a submucosal tumor in the upper deoxidation tract during routine gastroscopy, which was further confirmed by ultrasound gastroscopy. The location, size and nature of SMTs were determined. Anesthesia was evaluated and general anesthesia was performed with air tube intubation. In our hospital Endoscopic surgery (including ESD, ESE, EFR, STE R); Have complete clinical data and follow-up information; past No other malignancies. | NA |
| Zou Huan | 2022 | Retrospective study | Gastric stromal tumor | NA | Incision was made 4 to 5 cm from the proximal end of the tumor The mucosal layer, with an opening size of 1.5 ~ 2.0 cm, is located in the submucosa and muscle layer A longitudinal tunnel was established directly to the tumor, and the whole incision was performed after exposing the lesion Tumor, argon plasma coagulation (argon plasma coagulation, APC) (Olympus, FD-410LR) and hot biopsy forceps were treated inside the tunnel The mucosal incision was closed with a metal clamp. All patients Gastroscopy was performed at 1, 6 and 12 months after treatment, and CT was regularly reviewed All patients were followed up for 1 year. Record follow-up information Fella." | Dual Knife (Olympus, KD-650L) is used The tumor basal margin was marked with an injection needle (Olympus, NM-200L-0432) Mix diluent (indigo carmine: adrenaline: normal saline =3 ~5 ml∶1 ml∶100 ml) were injected into the surrounding mucosa at multiple points The mucous membrane around the tumor base was cut with a Dual Knife and peeled beneath the tumor The submucosa was removed, the tumor was completely removed, and finally the wound was electrocoagulated. In EFR group, ESD was used to separate the tumor from the surrounding to the serous membrane Use IT Knife (Olympus, KD-611L) or Dual Knife to cut the part Complete resection of serous membranes and tumors caused by iatrogenic gastric perforation using titanium Clamp, nylon rope and titanium clamp (Micro-Tech Co, ROCC-D-26-165) Or Over-scope clip (OTSC) Wait to close the wound. | ① Preoperative gastroscopy and endoscopic ultrasound indicated the presence of gastric submucosal tumors Acoustic endoscopy identified the lesion level . Preoperative CT evaluation showed no metastatic lesions; ③ The maximum tumor diameter was 3.0 ~ 5.0 cm. | ① Simultaneous presence of esophageal or duodenal lesions; ② There are multiple gastric diseases The kitchen stove. |
| Tu Sufang | NA | Retrospective study | Submucosal tumors (SMT) | NA | NA | NA | NA | NA |
| Lin Liangdou | 2018 | Retrospective study | Upper gastrointestinal submucosal tumors | Plasmatoma, leiomyoma, lipoma, cyst, hemangioma, neuroendocrine tumor. | ① Lesion location: Install a transparent tube at the head end of the electronic gastroscope, search the cavity and look for swelling step by step Tumor, and accurate location. ② To create a submucosal tunnel: a linear distance of 3 ~ 5cm from the cavity probe to the tumor's hearth side A transverse incision, longitudinal incision or oblique incision is made under the digestive tract mucosa. The essence of the operation method is to locally inject indigo carmine mixture with an injection needle to make the local mucosal layer fully raised and used A 1.5 ~ 2.0cm incision was made in the mucosal layer by electroknife, and the submucosa and musculi propria were gradually separated A longitudinal tunnel is established between the two, and if there is bleeding in the tunnel, hot biopsy forceps or argon knife can be used Stop the bleeding. ③ The tumor was completely removed and resected under the direct vision of the electronic gastroscope: the tumor was seen after the tunnel was fully established The tumor body is gradually separated from the surrounding tissue by HOOK knife, IT knife or Hybird knife The tumor body and its envelope were completely removed to avoid damage to the serous membrane and mucosal surface of the digestive tract The destruction. For submucosal tumors, if the tumor body is closely connected with the serous membrane, electrotome can be used The serous membrane is cut along the perimeter of the tumor to completely remove the tumor if this results in more severe gas Abdominal, abdominal puncture needle can be used to puncture the right lower abdomen to continuously exhaust to reduce abdominal pressure, but also to avoid tumor Falling into the abdominal cavity and bleeding at the incisal margin of the tumor. ④ Closure of the tunnel mouth: after complete resection of the tumor, if there is a bleeding focus or visible small blood vessels, you can After treatment with APC or hot biopsy forceps, the gas and liquid in the tunnel were fully attracted by electronic gastroscope Exit the submucosal tunnel, and finally use 4 to 6 metal titanium clips to completely clamp the mucosal incision. | ① Determine the scope and depth of the lesion: Before ESD, conventional electronic gastroscopy and ultrasound are required Endoscopic examination is mainly for the location, size and shape of the lesion, the origin level and nature of the lesion For more detailed understanding, so that ESD can be carried out smoothly. ② Labeling: Use conventional electronic gastroscopy and endoscopic ultrasonography to fully understand the lesions and perform the lesions The specific operation method is to use APC with the aid of electronic gastroscope and tip transparent tube Marks were made about 5-10mm away from the lesion margin. ③ Submucosal liquid injection: The main purpose of submucosal liquid injection is to fully raise the lesion It is separated from the muscle layer to achieve the purpose of complete resection of the lesion. The essence of the specific operation method is in disease Multiple submucosal fluids (normal saline + indigo carmine + epinephrine) were administered at the lateral margin of the focal marker injection. Incision: mucosal incision should generally be carried out at the distal end of the lesion, such as the difficulty of incision is relatively large Consider flipping endoscopy. The essence of the specific operation method is to first go along the mark point or outside the mark point Part of the mucosa around the lesion was incised by ESD electrotome at the side edge, and then ESD was used again The submucosa of the incision is gradually and deeply cut by the special electric knife to achieve the incision around the whole Mucosal purpose. ⑤ Submucosal dissection: whether the lifting of the lesion is good is related to the effectiveness of submucosal dissection Therefore, it is necessary to make adequate preparation and judgment before performing lesion dissection. The stripping process follows With the passage of time, the fluid injected under the mucosa (normal saline + indigo carmine + adrenaline mixture) It will be slowly absorbed by the surrounding tissue, so in order to fully lift the lesion, ESD can be performed on the submucosal Tissue repeated fluid injection. ⑥ In the process of peeling the lesion using ESD special electrotome, sometimes it is difficult to encounter the lesion In the case of exposure and unclear field of view under the microscope, in order to fully expose the endoscopic field of view for better access During ESD, consider using the transparent riser of the inner lens to push away the connective tissue of the submucosa. And peel off In the process, another key point of endoscopic operation is that the lesion needs to be performed along the basal tangent direction of the lesion Peeling can be done by pulling a mirror or rotating a mirror. 7 Wound treatment: After the lesion is completely removed by ESD special electroknife, the wound needs to be cleaned To prevent bleeding and other complications. In general, visible blood vessels on the wound can help Prophylactic hemostasis with hot biopsy forceps; Use hemostatic forceps, APC and other special tools The site of bleeding is treated with electric coagulation, or in the case that the ordinary treatment method is not ideal Clamp with metal titanium clamp; For deep lesions, ESD special electroknife peeling layer is more If there are cracks in the deep, peeled muscle layer, metal titanium clamping should be used." | ① The length and diameter of the tumor treated by STER or ESD should be ≤4cm, and the tumor site should occur in food Ducts, gastric fundus and cardia; ② All the included cases received electronic gastroscopy, endoscopic ultrasonography or CT auxiliary examination The tumor is submucosal, does not invade the digestive tract, the tumor boundary is clear, and there is no local or distant metastasis. No lymph node metastasis; ③ All patients were able to tolerate general anesthesia and had no other contraindications with anesthesia. | ① Patients with poor coagulation function and bleeding tendency; ② Psychological can not accept or physical can not tolerate general anesthesia surgery; ③ Patients with severe hypertension; ④ important organs (heart, lung, kidney, brain, etc.) function failure can not tolerate surgery; ⑤ Other problems can not be operated on, such as pregnancy, menstruation, etc. |
| Liu Ying | 2014 | Retrospective study | Submembranous tumors of the upper digestive tract | Leiomyoma, lipoma, ectopic pancreas, hyperplastic polyp, or borderline stromal tumor | Locate and inject an endoscope with a clear cap on the top into the esophagus or stomach through the mouth to locate the lesion, The solution mixed with methylene blue was injected repeatedly on the oral side at a linear distance from the lesion In the submucosa, the purpose is to raise the point membrane and separate it from the solid muscle layer; Slit: A longitudinal slit is cut on the surface of the injection point film, usually under the exposure point film Layer; Construction path: Push the inner lens end into the submucosal layer, between the point membrane layer and the solid muscle layer, and use Knife, knife, knife along the lower layer of the membrane to the lesion site, while peeling submucosal injection, Until the lesion site is reached, the tunnel from the wound to the lesion is established with the gap between the point membrane and the muscular layer Tao, expose the swelling. Under the condition of direct vision in the tunnel, the pathological changes were removed and the measurements were sent to the pathology Histochemical examination of the epidemic. Carefully observe the wound for blood and perforation, and finally close the opening with a metal titanium clip. In both cases, adrenalin saline mixture, hot biopsy forceps, argon ion coagulation were used. If the intraoperative lesion is difficult to peel away, it can be used The loop applicator is used to remove the loop. After surgery, the wound usually does not need to be completely closed with metal clips If there is a perforation, it is generally closed with a metal titanium clip after the removal of the lesion, if the surgical wound is deep or normal The circumference is larger, and the wound surface can also be closed or reduced with metal titanium clips. Postoperative wound should be finished with metal clip Total closure to prevent food or liquid from entering the subsarcomial tunnel. After the operation, carefully observe the wound surface for blood and perforation, It can prevent the occurrence of subcutaneous emphysema, pneumothorax, pneumoperitoneum and mediastinum. | Marker: An endoscope with a clear cap on the top enters the food tube or stomach through the mouth to find the lesion site, first The circumference of the lesion was marked with a knife. Injection: Use an injection needle to inject methylene blue solution into the adhesive at multiple points along the marked perimeter Under the membrane, make the point membrane lift well; Incision: Arc incision along the marked points, knife, knife, knife coordinate with each other, The surface point film was removed and the pathological tissue under the point film was exposed. Stripping: the rational use of knives, knives, knives carefully strip the tissues and blood tubes around the tumor, In the process of exfoliation, the submucosal injection is gradually absorbed into the tissue, which requires repeated submembranous injection until the lesion is finally exfoliated and the tissue is measured and sent to pathology for pathological and immunohistochemical examination. | Tumor diameter ≤4cm Gastroscopy, ultrasonic endoscopy or other auxiliary examination indicated that the tumor boundary was clear, and did not infiltrate or invade outside the Xiaohua channel In organs and tissues, there was no thoracoabdominal metastasis and no signs of luteo metastasis. (All others can tolerate general anesthesia surgery Comorbidity | NA |
| Yuyong Tan | 2016 | Retrospective study | gastric GISTs | gastric GISTs | (a) submucosal injection at about 3–5 cm proximal to the SMT; (b) a longitudinal mucosal incision was made to create the tunnel entry; (c) a submucosal tunnel was made between the submucosal and the MP layers; (d) dissection of the tumor with an insula tion-tip knife, a hybrid knife or a dual knife; and (e) close the mucosal entry. | (a) sub mucosal injection and precutting the mucosal and submu cosal layer around the lesion; (b) circumferential incision as deep as the MP layer around the lesion by the ESD technique; (c) incision into the serosal layer around the lesion; (d) full-thickness resection of the tumor including the serosal layer; (e) closing the gastric-wall defect with metallic clips. A gastric tube was placed after the proce dure. | (a) presence of gastric SMTs originating from the muscularis propria (MP) layer confirmed by EGD, EUS andNAor computerized tomography (CT), and the final diagnosis was GIST; (b) EUS shows no high-risk features of malignancy, such as irregular border, internal heterogeneity and heteroge neous enhancement; (c) no signs of metastasis or invasion outside the gastrointestinal tract during CT examination; (d) patient consent to undergo an STER or EFTR procedure at our hospital. | Those patients who could not tolerate anesthesia and those with severe cardiopulmonary disease or blood coagulation disorders (international normalized ratio [2.0, platelet count \100 000NAmm3 ) were excluded from the study. |
