# Supplementary material for: The efficacy and safety of submucosal tunnel endoscopic resection for the treatment of upper gastrointestinal submucosal tumors: a systematic review and meta-analysis
Source: Front Oncol. 2025 Aug 7;15:1584205. doi: 10.3389/fonc.2025.1584205 (PMC12367509; doi:10.3389/fonc.2025.1584205)
Supplement: Supplementary file 2 [file Table2.docx]

| **Supplementary Table 2 quality Assessment (Critical Appraisal Skills Programme(CASP)** | | | | | | | | | | | | | |
| --- | --- | --- | --- | --- | --- | --- | --- | --- | --- | --- | --- | --- | --- |
| First author | Year of publication | 1. Did the study address a  clearly focused issue? | 2. Did the authors use an  appropriate method to  answer their question? | 3. Were the cases recruited in  an acceptable way? | 4. Were the controls selected in  an acceptable way? | 5. Was the exposure accurately  measured to minimise bias? | 6. (a) Aside from the  experimental intervention,  were the groups treated  equally? | 6. (b) Have the authors taken  account of the potential  confounding factors in the  design and/or in their  analysis? | 7. How large was the treatment effect? | 8. How precise was the estimate of the treatment  effect? | 9. Do you believe the results? | 10. Can the results be applied  to the local population? | 11. Do the results of this study  fit with other available  evidence? |
| Michel Kahaleh | 2022 | yes | yes | yes | yes | Can't tell | yes | no | The article did not mention the effect size and confidence interval, and mainly used some objective data such as complete resection rate, operation time and hospital stay to evaluate the therapeutic effect. | The study found that the treatment was effective, but the article did not mention confidence intervals. The small sample size of the study may have limited the accuracy of the estimates, and further studies with larger sample sizes are needed to confirm these findings. | yes | yes | yes |
| Philip Wai Yan Chiu | 2022 | yes | yes | yes | yes | Can't tell | yes | no | The article did not mention the effect size and confidence interval, and mainly used some objective data such as complete resection rate, operation time and hospital stay to evaluate the therapeutic effect. | The study found that the treatment was effective, but the article did not mention confidence intervals. The small sample size of the study may have limited the accuracy of the estimates, and further studies with larger sample sizes are needed to confirm these findings. | yes | yes | yes |
| Luo Yingshu | 2021 | yes | yes | yes | yes | yes | yes | yes | The article did not mention the effect size and confidence interval, and mainly used some objective data such as complete resection rate, operation time and hospital stay to evaluate the therapeutic effect. | The study found that the treatment was effective, but the article did not mention confidence intervals. The small sample size of the study may have limited the accuracy of the estimates, and further studies with larger sample sizes are needed to confirm these findings. | yes | yes | yes |
| Zou Huan | 2022 | yes | Can't tell | yes | Can't tell | no | yes | no | The article did not mention the effect size and confidence interval, and mainly used some objective data such as complete resection rate, operation time and hospital stay to evaluate the therapeutic effect. | The study found that the treatment was effective, but the article did not mention confidence intervals. The small sample size of the study may have limited the accuracy of the estimates, and further studies with larger sample sizes are needed to confirm these findings. | yes | yes | yes |
| Tu Sufang | NA | yes | Can't tell | Can't tell | Can't tell | Can't tell | Can't tell | Can't tell | The article did not mention the effect size and confidence interval, and mainly used some objective data such as complete resection rate, operation time and hospital stay to evaluate the therapeutic effect. | The study found that the treatment was effective, but the article did not mention confidence intervals. The small sample size of the study may have limited the accuracy of the estimates, and further studies with larger sample sizes are needed to confirm these findings. | yes | yes | yes |
| Lin Liangdou | 2018 | yes | yes | yes | yes | Can't tell | yes | yes | The article did not mention the effect size and confidence interval, and mainly used some objective data such as complete resection rate, operation time and hospital stay to evaluate the therapeutic effect. | The study found that the treatment was effective, but the article did not mention confidence intervals. The small sample size of the study may have limited the accuracy of the estimates, and further studies with larger sample sizes are needed to confirm these findings. | yes | yes | yes |
| Liu Ying | 2014 | yes | yes | Can't tell | Can't tell | Can't tell | yes | yes | The article did not mention the effect size and confidence interval, and mainly used some objective data such as complete resection rate, operation time and hospital stay to evaluate the therapeutic effect. | The study found that the treatment was effective, but the article did not mention confidence intervals. The small sample size of the study may have limited the accuracy of the estimates, and further studies with larger sample sizes are needed to confirm these findings. | yes | yes | yes |
| Yuyong Tan | 2016 | yes | yes | yes | yes | Can't tell | Can't tell | Can't tell | The article did not mention the effect size and confidence interval, and mainly used some objective data such as complete resection rate, operation time and hospital stay to evaluate the therapeutic effect. | The study found that the treatment was effective, but the article did not mention confidence intervals. The small sample size of the study may have limited the accuracy of the estimates, and further studies with larger sample sizes are needed to confirm these findings. | yes | yes | yes |
